# Contribution of STAT1 to innate and adaptive immunity during type I interferon-mediated lethal virus infection

**DOI:** 10.1371/journal.ppat.1008525

**Published:** 2020-04-20

**Authors:** So Ri Jung, Thomas M. Ashhurst, Phillip K. West, Barney Viengkhou, Nicholas J. C. King, Iain L. Campbell, Markus J. Hofer

**Affiliations:** 1 School of Life and Environmental Sciences, The University of Sydney, Sydney, Australia; 2 The Marie Bashir Institute for Infectious Diseases and Biosecurity, the Charles Perkins Centre and the Bosch Institute, The University of Sydney, Sydney, Australia; 3 Sydney Cytometry Core Facility, The University of Sydney and Centenary Institute, Sydney, Australia; 4 Department of Pathology, School of Medical Sciences, Sydney Medical School, The University of Sydney, Sydney, Australia; Emory University, UNITED STATES

## Abstract

Signal transducers and activators of transcription (STAT) 1 is critical for cellular responses to type I interferons (IFN-Is), with the capacity to determine the outcome of viral infection. We previously showed that while wildtype (WT) mice develop mild disease and survive infection with lymphocytic choriomeningitis virus (LCMV), LCMV infection of STAT1-deficient mice results in a lethal wasting disease that is dependent on IFN-I and CD4^+^ cells. IFN-Is are considered to act as a bridge between innate and adaptive immunity. Here, we determined the relative contribution of STAT1 on innate and adaptive immunity during LCMV infection. We show that STAT1 deficiency results in a biphasic disease following LCMV infection. The initial, innate immunity-driven phase of disease was characterized by rapid weight loss, thrombocytopenia, systemic cytokine and chemokine responses and leukocyte infiltration of infected organs. In the absence of an adaptive immune response, this first phase of disease largely resolved resulting in survival of the infected host. However, in the presence of adaptive immunity, the disease progressed into a second phase with continued cytokine and chemokine production, persistent leukocyte extravasation into infected tissues and ultimately, host death. Overall, our findings demonstrate the key contribution of STAT1 in modulating innate and adaptive immunity during type I interferon-mediated lethal virus infection.

## Author summary

The mammalian immune system is divided into innate and adaptive immunity. In response to harmful agents, innate immunity acts first, followed by late-acting, specialized, adaptive immunity. Type I interferons (IFN-Is) are important means of communication between innate and adaptive immunity. IFN-Is mediate their effects via a number of signaling molecules, principally including signal transducers and activators of transcription 1 (STAT1). The importance of STAT1 to the immune response is evident from our previous finding that mice deficient in STAT1 develop a lethal, host immunity-mediated disease following infection with the otherwise harmless lymphocytic choriomeningitis virus (LCMV). In the present study, we characterized the role of STAT1 in protecting against harmful host immune responses against LCMV. We report that STAT1 plays a significant role in lessening both the early, inflammatory responses of innate immunity and the sustained, destructive actions of adaptive immunity. These findings exemplify the extent of STAT1’s role as a key immune response modulating factor.

## Introduction

Type I interferons (IFN-Is) are a large family of potent antiviral and immunomodulatory cytokines that includes multiple IFN-α subtypes, IFN-β and other single gene products. IFN-Is play crucial, antiviral and immunomodulatory roles, activating and regulating cells of both the innate and adaptive immune compartments. For example, IFN-I signaling increases degranulation of neutrophils [1] and mediates dendritic cell (DC) maturation and activation [2–4]. Furthermore, IFN-Is orchestrate CD4^+^ T cell activation and differentiation [5, 6]. They also directly promote the clonal expansion, survival, production of IFN-γ and development of cytotoxic functions of anti-viral CD8^+^ T cells [7].

Signal transducers and activators of transcription 1 (STAT1) is a critical component of IFN-I signaling [8, 9]. When IFN-Is bind to the IFN-α/β receptor 1 and 2 subunits, STAT1 and STAT2 are activated and subsequently form a trimolecular complex with interferon regulatory factor 9, termed interferon-stimulated gene factor 3 [10–12]. This complex regulates the expression of several hundred interferon-regulated genes orchestrating the antiviral host response.

Lymphocytic choriomeningitis virus (LCMV) is a prototypical mammarenavirus [13, 14]. LCMV infection of mice has been one of the most widely used model systems to study viral pathogenesis and antiviral immune mechanisms [15, 16], owing in part to the non-cytopathic nature of the virus [17]. Accordingly, following intraperitoneal (i.p.) infection, immunocompetent mice develop mild or no signs of disease and between days 8–14 postinfection, IFN-I-dependent CD8^+^ T cell expansion and activation mediates viral clearance [18–21]. By contrast, similarly infected STAT1-deficient (STAT1 KO) mice develop a lethal, immunopathological disease in an IFN-I-dependent, IFN-γ-independent manner [22, 23]. We previously found that IFN-I signaling mediates early and pronounced decline in physical condition including rapid weight loss of LCMV-infected STAT1 KO mice, suggesting a key role for the early, innate immune response in contributing to the disease [22]. While antibody-mediated depletion of CD8α^+^ cells extended the survival, it did not prevent the death of these mice, whereas antibody-mediated depletion of CD4^+^ cells provided complete protection against lethality [23]. These findings suggest that both innate and adaptive immunity contribute to the lethal disease in the STAT1 KO mice. Here, we aimed to determine the contribution of STAT1 in regulating innate versus adaptive immunity in this model of IFN-I-mediated, lethal antiviral response.

## Results

### The innate immune compartment mediates early and rapid weight loss while adaptive immunity is required for lethality in LCMV-infected STAT1 KO mice

**Fig 1:**
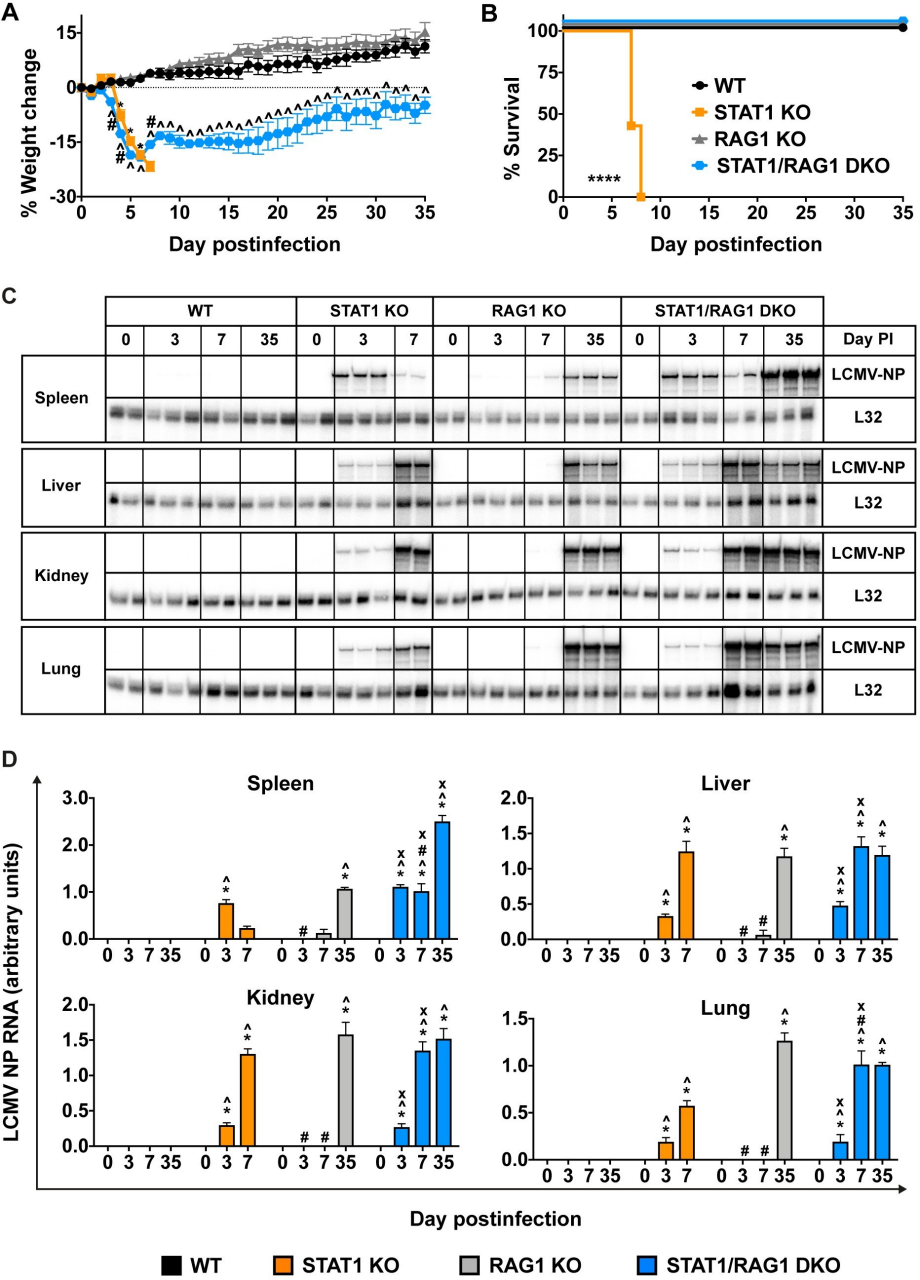
RAG1-deficiency protects STAT1 KO mice from lethal LCMV infection but has little impact on the control of virus spread and replication. WT (n = 21), STAT1 KO (n = 28), RAG1 KO (n = 22) and STAT1/RAG1 DKO (n = 21) mice were infected with 500 pfu of LCMV-Arm i.p., as described in Materials and Methods. (A) Weight change, combined data from 4 independent experiments. (B) Survival outcome, combined data from 4 independent experiments. For significance (Log-rank test): ****, P<0.0001 compared with WT mice. Data are shown as means ± SEM. For significance (one-way ANOVA with Tukey post-test): *, P<0.05 for STAT1 KO mice compared with WT mice; ^, P<0.05 for STAT1/RAG1 DKO mice compared with WT mice; #, P<0.05 for STAT1/RAG1 DKO mice compared with STAT1 KO mice. RPA was performed on total RNA (20 μg) from spleen, liver, kidney and lung of mock- and LCMV-infected WT, STAT1 KO, RAG1 KO and STAT1/RAG1 DKO mice to determine the level of LCMV-NP RNA. Representative autoradiograph (n = 2–3 per time-point per genotype) is shown (C) and values from densitometric analysis were normalized to the corresponding L32 loading control and the combined results from two independent experiments (n = 4–6 per time-point per genotype) are expressed as the mean ± SEM (D). For significance (one-way ANOVA with Tukey post-test): *, P<0.05 compared with mock-infected mice; ^, P<0.05 compared with WT mice at the respective time-points; #, P<0.05 compared with STAT1 KO mice at the respective time-points; x, P<0.05 compared with RAG1 KO mice at the respective time-points. Day 0 postinfection denotes the mock-infected mice.

To study the role of adaptive immune cells in LCMV-induced lethal disease of STAT1-deficient mice, we interbred recombination activation gene-1 knock-out (RAG1 KO) mice that lack mature T and B cells [24], with STAT1 KO mice to generate STAT1/RAG1 DKO mice. Following i.p. inoculation with LCMV, wildtype (WT) and RAG1 KO mice showed little to no signs of disease at any observation time postinfection (Fig 1A). By contrast, similarly infected STAT1 KO and STAT1/RAG1 DKO mice had rapid weight loss accompanied by other signs of disease such as hunched posture, reduced activity and ruffled fur from day 4 postinfection (Fig 1B). STAT1 KO mice continued to lose weight and decline in condition and had to be euthanized between days 7 and 8 postinfection in accordance with animal welfare requirements, having lost up to 20% of their initial body weight (Fig 1A and 1B). By contrast, similarly infected STAT1/RAG1 DKO mice did not reach the critical weight loss limit or the clinical score for humane endpoint, and from day 7 postinfection, gained weight, showed increased activity and regained up to 95% of original body weight by day 35 postinfection (Fig 1A).

Although the control and eventual clearance of LCMV infection is crucially dependent on CD8^+^ T cells in WT mice [20, 21], the role of adaptive immunity in the control of viral replication and spread in STAT1 KO mice is unknown and was investigated next (Fig 1C and 1D). To assess virus load, we performed RNase Protection Assay (RPA) for LCMV-NP RNA, which we had previously shown to correlate well with infectious virus load during acute infection [23]. In agreement with our previous findings [22, 23], LCMV-NP RNA was undetectable in all organs tested in infected WT mice. However, in RAG1 KO mice, while LCMV-NP RNA was not detectable during early stages of infection (day 3 postinfection), it was present at low levels in spleen and liver on day 7 postinfection and was detectable at high levels in all organs at day 35 postinfection, confirming the need for T cells for virus elimination. By contrast, LCMV-NP RNA was detectable in all organs of infected STAT1 KO and STAT1/RAG1 DKO mice from day 3 postinfection (Fig 1C and 1D) and remained high in STAT1/RAG1 DKO mice at day 35 postinfection. Taken together, these findings indicate that innate immunity mediated the early and rapid weight loss, while adaptive immunity was necessary for lethality in STAT1 KO mice following LCMV infection. Notably, RAG1 KO mice exhibited a remarkable, STAT1-dependent capacity to control LCMV infection initially but this was ineffective as infection progressed.

### The combined actions of innate and adaptive immunity mediate systemic neutrophilia, but innate immunity is responsible for thrombocytopenia in LCMV-infected STAT1 KO mice

**Fig 2:**
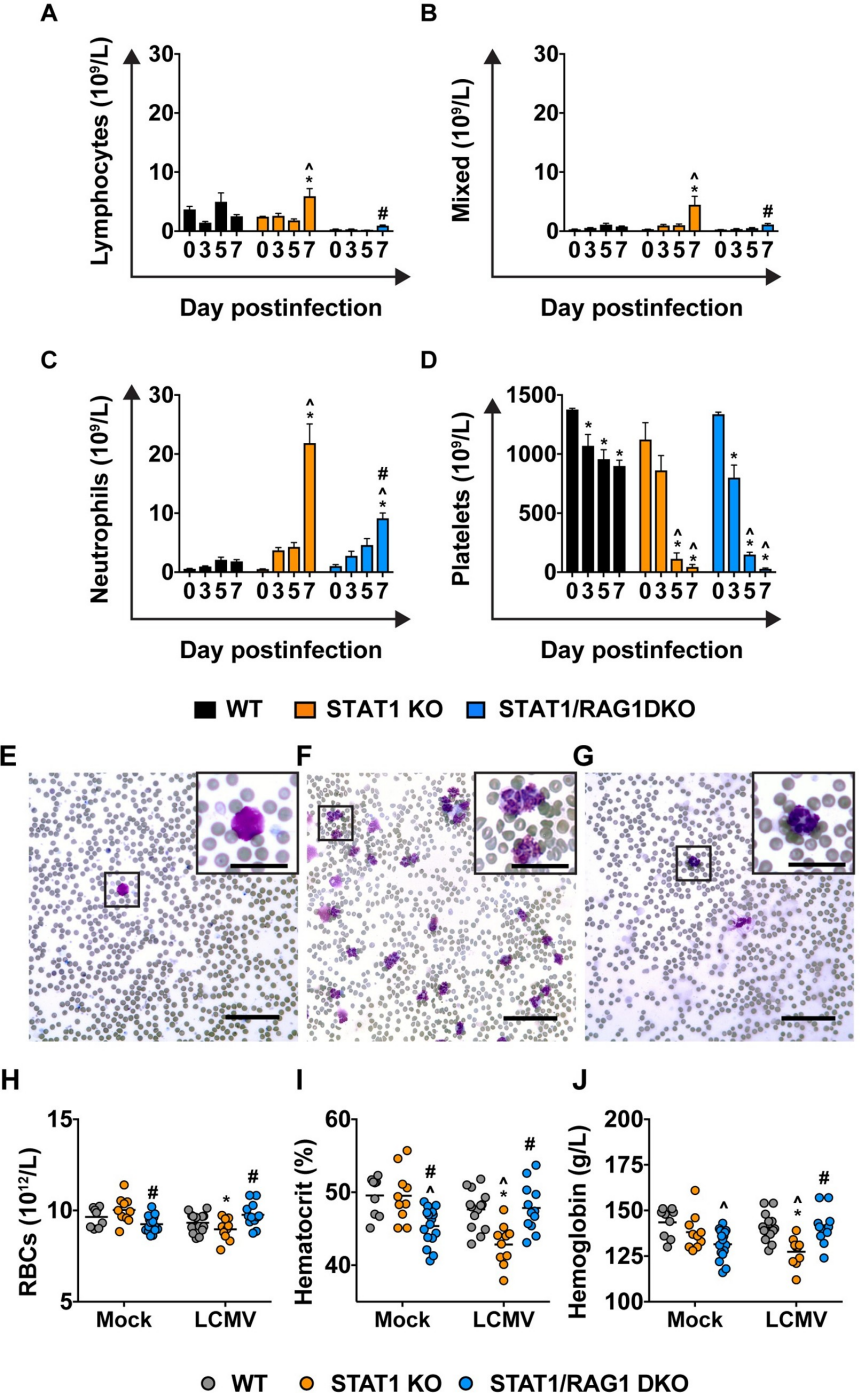
Combined actions of innate and adaptive immunity mediate systemic neutrophilia and mild anemia, but innate immunity mediates thrombocytopenia in LCMV-infected STAT1 KO mice. Number of lymphocytes (A), mixed leukocytes (B), neutrophils (C) and platelets (D) in peripheral blood of mock- and LCMV-infected WT, STAT1 KO and STAT1/RAG1 DKO mice, as determined by Sysmex XP-100 hematoanalyzer. The hematoanalyzer cannot distinguish monocytes, eosinophils and basophils and displays the total number of the three cell types as ‘mixed leukocytes’. Day 0 postinfection denotes mock-infected group. Diff-Quik-stained blood smears of LCMV-infected (day 7 postinfection) WT (E), STAT1 KO (F) and STAT1/RAG1 DKO (G) mice. Scale bar = 62.5 μm. Insets depict higher magnification of circulating leukocytes (scale bar = 25 μm). Number of red blood cells (RBCs) (H), hematocrit (%) (I) and concentration of hemoglobin (J) in peripheral blood of mock- and LCMV-infected (day 7 postinfection) WT, STAT1 KO and STAT1/RAG1 DKO mice, as determined by Sysmex XP-100 hematoanalyzer. Line indicates mean. For significance (two-way ANOVA with Tukey post-test): *, P<0.05 compared with mock-infected mice; ^, P<0.05 compared with WT mice at the respective infection group; #, P<0.05 compared with STAT1 KO mice at the infection group. Sample size was as follows: WT mice mock-infected (n = 9), WT mice day 3 postinfection (n = 6), WT mice day 5 postinfection (n = 6), WT mice day 7 postinfection (n = 14), STAT1 KO mice mock-infected (n = 10), STAT1 KO mice day 3 postinfection (n = 6), STAT1 KO mice day 5 postinfection (n = 6), STAT1 KO mice day 7 postinfection (n = 10), STAT1/RAG1 DKO mice mock-infected (n = 16), STAT1/RAG1 DKO mice day 3 postinfection (n = 6), STAT1/RAG1 DKO mice day 5 postinfection (n = 5) and STAT1/RAG1 DKO mice day 7 postinfection (n = 12).

Arenavirus infection can cause severe hemorrhagic diseases in humans [25]. Similarly, in mice, various models described systemic LCMV infection inducing thrombocytopenia and vascular leakage leading to death [26–28]. To investigate whether hemorrhagic disease contributes to lethality in LCMV-infected STAT1 KO, we determined the quantitative changes in the leukocyte subsets, platelets, red blood cells (RBCs), % hematocrit and hemoglobin level in the peripheral blood of these mice (Fig 2). In mock-infected WT, STAT1 KO and STAT1/RAG1 DKO mice, no significant differences in the number of leukocytes and thrombocytes were detected, apart from an absence of lymphocytes and lower RBCs, % hematocrit and hemoglobin levels in the STAT1/RAG1 DKO mice (Fig 2A and 2H–2J). Following infection, WT mice showed no significant increase in the number of leukocytes in peripheral blood (Fig 2A–2C). By contrast, there was a significant increase of all leukocyte subsets, especially neutrophils, in similarly infected STAT1 KO mice. STAT1/RAG1 DKO mice had a significant and progressive increase in the number of neutrophils but not monocytes, eosinophils and basophils. Further, neutrophil numbers were significantly higher in STAT1 KO mice when compared with STAT1/RAG1 DKO mice on day 7 postinfection with LCMV (Fig 2C).

Platelet numbers progressively decreased in WT mice following infection and by day 7 postinfection, were 75% of the original level (Fig 2D). In comparison, STAT1 KO and STAT1/RAG1 DKO mice showed a significant decline in platelet numbers on day 5 and by day 7 postinfection were less than 5% of the original level (Fig 2D). Blood smears showed no apparent platelet clumping excluding the possibility of pronounced pseudo-thrombocytopenia (Fig 2E–2G). There were no significant changes in RBC numbers, % hematocrit and hemoglobin level in WT mice on day 7 postinfection (Fig 2H–2J). By contrast, there was a significant decrease in these parameters in similarly infected STAT1 KO mice, although it did not exceed 15%. Similar to WT mice, there were no significant changes in the number of RBCs, hematocrit and hemoglobin level in STAT1/RAG1 DKO mice on day 7 postinfection (Fig 2H–2J). Taken together, our findings indicated that the adaptive immune compartment was required for excessive systemic neutrophilia in STAT1 KO mice following LCMV infection. Moreover, our findings showed that innate immune cells mediated thrombocytopenia whereas adaptive immune cells mediated mild anemia in infected STAT1 KO mice. Although previous reports linked thrombocytopenia to hemorrhagic disease in systemic LCMV infection (e.g. [28]), we only observed mild signs of anemia in these mice.

### Adaptive immune cells amplify granulocyte-skewed leukocyte infiltration in peripheral organs of LCMV-infected STAT1 KO mice

**Fig 3:**
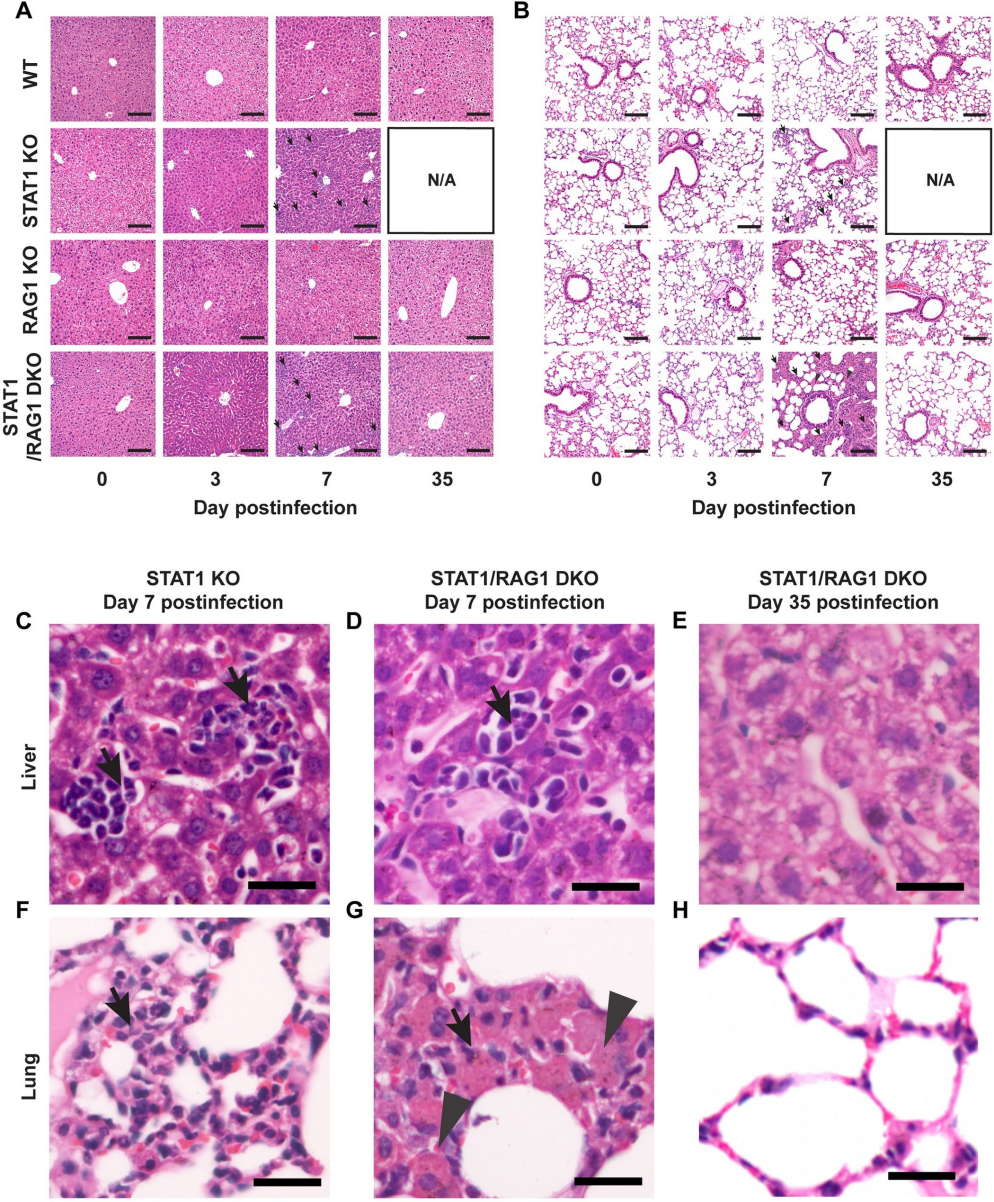
Innate immunity mediates gross tissue pathology in LCMV-infected STAT1 KO mice. Representative H&E images of liver (A, C-E) and lung (B, F-H). Scale bar = 125 μm. Higher magnification of representative H&E images (C-H) of liver (C-E) and lung (F-H) of STAT1 KO mice on day 7 postinfection (C and F), STAT1/RAG1 DKO mice on day 7 postinfection (D and G) and STAT1/RAG1 DKO mice on day 35 postinfection (E and H). Scale bar = 25 μm. Representative images from 3 independent experiments are shown. Arrows = infiltrating leukocytes. Arrowheads = thickened basement membrane. No images were collected for LCMV-infected STAT1 KO mice on day 35 postinfection as none survived.

We previously showed that LCMV-infected STAT1 KO mice have extensive immunopathology in peripheral organs, including a large number of infiltrating leukocytes and necrotic foci [22, 23]. To determine the contribution of adaptive immune cells in this pathology, histological examination was performed on the liver and lung of LCMV-infected WT, RAG1 KO, STAT1 KO and STAT1/RAG1 DKO mice (Fig 3). There were no observable differences in tissue morphology in the organs of mock-infected mice independent of the genotype. Following LCMV infection, little to no pathological changes were observed in the liver and lung of WT and RAG1 KO mice (Fig 3A and 3B). Although the same was true for STAT1 KO and STAT1/RAG1 DKO mice on day 3 postinfection, on day 7 postinfection, pronounced pathological changes were observed in both liver and lung, including an increased number of infiltrating leukocytes in these organs (Fig 3C, 3D, 3F and 3G). In particular, in the lungs of LCMV-infected STAT1 KO and STAT1/RAG1 DKO mice, a thickening of the basement membrane of bronchial epithelial cells was evident (Fig 3F and 3G). This was more severe in STAT1/RAG1 DKO mice when compared with STAT1 KO mice on day 7 postinfection (Fig 3F and 3G). However, by day 35 postinfection, STAT1/RAG1 DKO mice had a resolution of these pathological changes in both organs, showing no observable differences when compared with the mock-infected mice (Fig 3E and 3H). Notably, no overt bleeding was observed in the organs of LCMV-infected STAT1 KO mice, contrary to the reports of hemorrhagic diseases in systemic LCMV infection (e.g. [28]).

**Fig 4:**
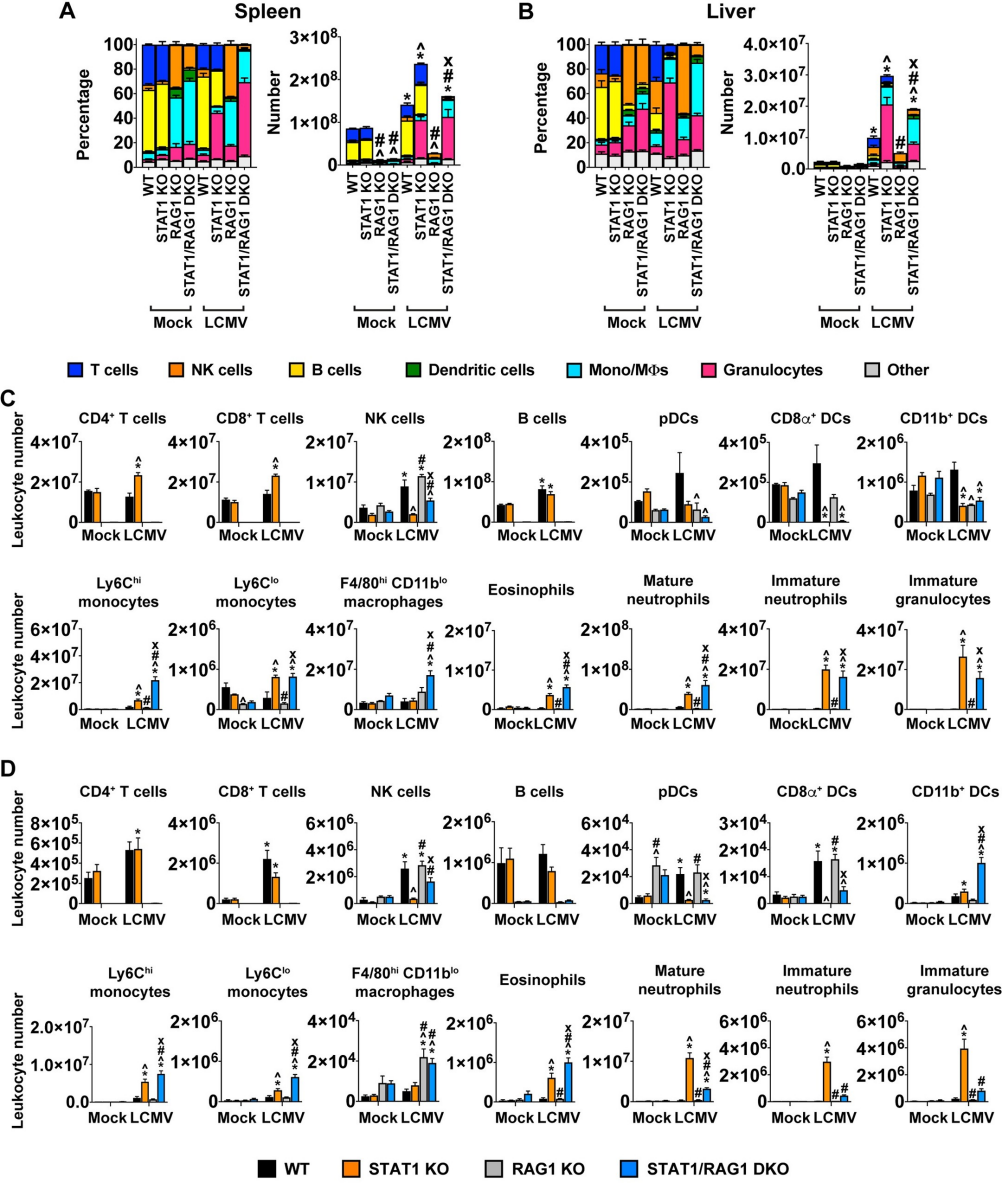
Granulocytes are the major group of leukocytes that infiltrate the peripheral tissues of LCMV-infected STAT1 KO mice in an adaptive-immunity-dependent manner. Leukocytes were isolated from the spleen and liver of mock- and LCMV-infected (day 7 postinfection) WT, STAT1 KO, RAG1 KO and STAT1/RAG1 DKO mice and flow cytometric analysis was performed, as described in Materials and Methods. Representative gating strategy is shown in S1 Fig. Percentage and total number of leukocytes in (A) spleen and (B) liver. Number of specific leukocyte population in whole spleen (C) and whole liver (D). For significance (two-way ANOVA with Tukey post-test): *, P<0.05 compared with uninfected mice; ^, P<0.05 compared with WT mice at the respective time points; #, P<0.05 compared with STAT1 KO mice at the respective time points; x, P<0.05 compared with RAG1 KO mice at the respective time points. Representative data of two independent experiments is shown (n = 3 for mock-infected and n = 5 for LCMV-infected per genotype).

We next asked if there was a difference in the cellular composition of the infiltrates between LCMV-infected STAT1 KO and STAT1/RAG DKO mice. To address this, we employed flow cytometry to determine the number and nature of leukocyte subsets in the spleen and liver of mock- and LCMV-infected mice on day 7 postinfection. Apart from the expected absence of T and B cells in RAG1 KO and STAT1/RAG1 DKO mice and an increased number of plasmacytoid DCs (pDCs) in the liver of RAG1 KO mice, there was no significant difference in leukocyte numbers or composition in spleen and liver of mock-infected mice from different genotypes (Fig 4). Following LCMV infection, there was a significant increase of leukocytes in the liver of WT mice and both in the spleen and liver of STAT1 KO and STAT1/RAG1 DKO but not RAG1 KO mice (Fig 4A and 4B). Further, the number of infiltrating leukocytes in the liver was significantly greater in infected STAT1 KO mice than in the other genotypes (Fig 4B).

When individual leukocyte subsets were quantified in the spleen, WT mice had a significant increase of natural killer (NK) cells and B cells and RAG1 KO mice had a significant increase of NK cells only following LCMV infection (Fig 4C). By contrast, infected STAT1 KO mice showed a significant increase in CD4^+^ and CD8^+^ T cells, B cells, Ly6C^hi^ and Ly6C^lo^ monocytes, eosinophils, mature and immature neutrophils, and immature granulocytes, and a significant decrease of CD8α^+^ and CD11b^+^ DCs. STAT1/RAG1 DKO mice had a significant increase of Ly6C^hi^ and Ly6C^lo^ monocytes, F4/80^hi^ CD11b^lo^ macrophages, eosinophils, and mature and immature neutrophils and a significant decrease of CD8α^+^ and CD11b^+^ DCs. Notably, the numbers of Ly6C^hi^ monocytes, F4/80^hi^ CD11b^lo^ macrophages, eosinophils and mature neutrophils in STAT1/RAG1 DKO mice were significantly greater than in STAT1 KO mice following infection.

In the liver following infection, NK cells, pDCs, CD8α^+^ DCs and CD8^+^ T cells were significantly increased in WT mice and NK cells, as were CD8α^+^ DCs and F4/80^hi^ CD11b^lo^ macrophages in RAG1 KO mice (Fig 4D). In infected STAT1 KO mice, there was a significant increase of myeloid cells (monocytes, macrophages and granulocytes), while infected STAT1/RAG1 DKO mice had a significant increase of CD11b^+^ DCs, Ly6C^hi^ monocytes, Ly6C^lo^ monocytes, F4/80^hi^ CD11b^lo^ macrophages, eosinophils and mature neutrophils (Fig 4D). There was a significant decrease of pDCs in STAT1/RAG1 DKO mice following infection, the numbers similar to that in infected STAT1 KO mice. Notably, while the numbers of CD11b^+^ DCs, Ly6C^hi^ and Ly6C^lo^ monocytes, F4/80^hi^ CD11b^lo^ macrophages and eosinophils were significantly greater in STAT1/RAG1 DKO mice when compared with STAT1 KO mice, the numbers of mature and immature neutrophils and immature granulocytes were significantly greater in STAT1 KO mice than in STAT1/RAG1 DKO mice following infection.

Taken together, these findings indicate that while innate immunity caused gross leukocyte infiltration into the infected organs of LCMV-infected STAT1 KO mice, adaptive immunity was responsible for the excessive number of infiltrating neutrophils, consistent with the hematological findings.

### RAG1-deficiency does not impair IFN-I production in LCMV-infected STAT1 KO mice

**Fig 5:**
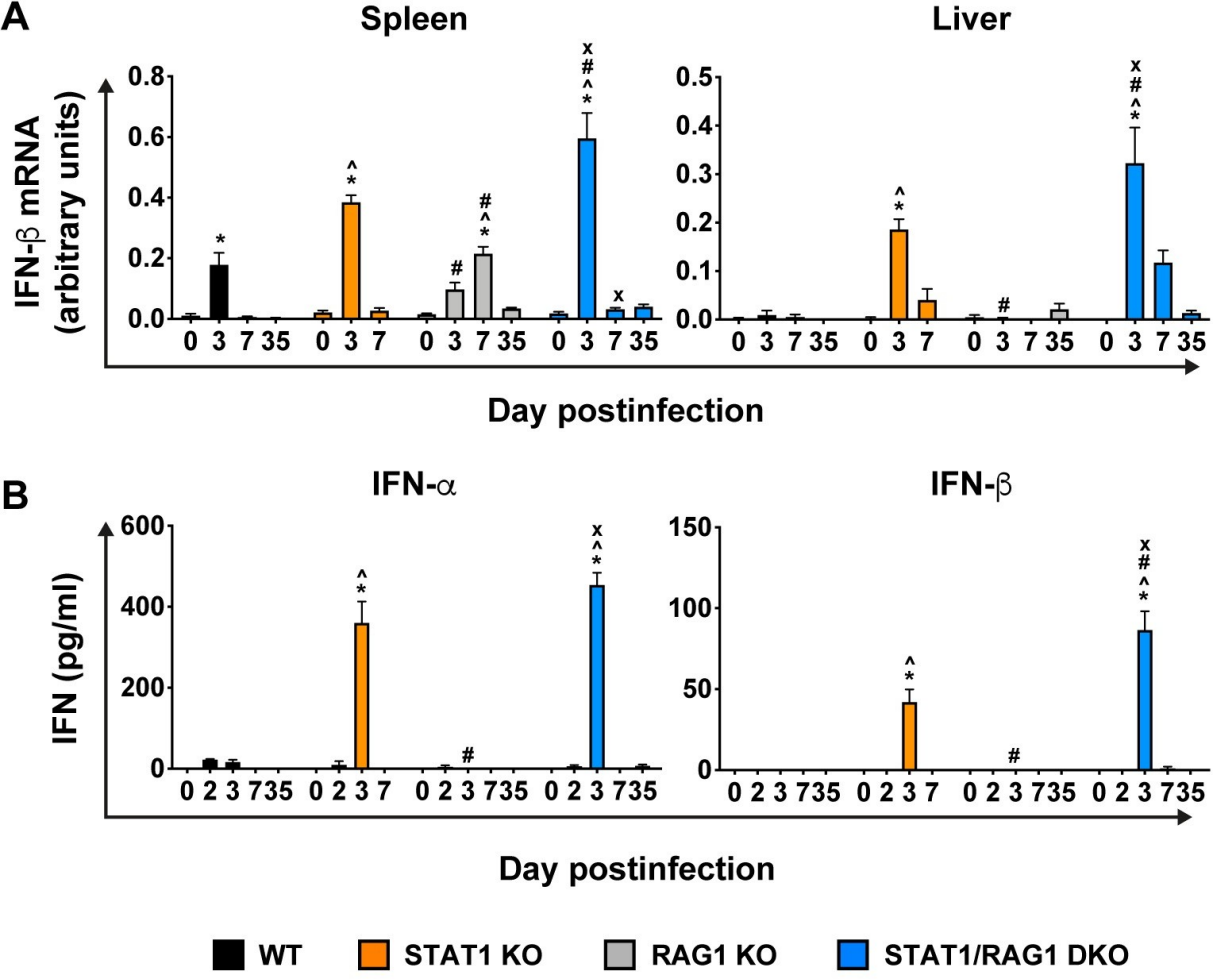
RAG1 deficiency does not impair IFN-I production in LCMV-infected STAT1 KO mice. RPA was performed on total RNA (30 μg) of spleen and liver of WT, STAT1 KO, RAG1 KO and STAT1/RAG1 DKO mice infected i.p. with LCMV, to determine the levels of IFN-β mRNA (n = 4–6 per time-point per genotype). Values from densitometric analysis were normalized to the corresponding L32 loading control and the combined results from two independent experiments were expressed as the mean ± SEM (A). IFN-α and -β ELISAs were performed on plasma of WT, STAT1 KO, RAG1 KO and STAT1/RAG1 DKO mice infected i.p. with LCMV (B). Combined data from two independent experiments is shown (n = 4–8 per time-point per genotype). For significance (one-way ANOVA with Tukey post-test): *, P<0.05 compared with uninfected mice; ^, P<0.05 compared with WT mice at the respective time-points; #, P<0.05 compared with STAT1 KO mice at the respective time-points; x, P<0.05 compared with RAG1 KO mice at the respective time-points. Day 0 PI denotes mock-infected mice.

It was reported previously that RAG1-deficient mice have impaired production of IFN-Is during LCMV infection due to disrupted splenic architecture [29]. Indeed, there was a lack of defined splenic architecture in both RAG1 KO and STAT1/RAG1 DKO mice (S2 Fig). Hence, as IFN-I production and signaling is necessary for the development of the lethal disease in LCMV-infected STAT1 KO mice [22], it remained possible that the survival of STAT1/RAG1 DKO mice following infection was due to impaired IFN-I production. To address this, we determined the level of IFN-β mRNA in spleen and liver and systemic levels of IFN-α and -β in LCMV-infected mice (Fig 5).

IFN-β mRNA was undetectable in the spleen and liver of all mock-infected mice, irrespective of genotype (Fig 5A). Following infection, IFN-β mRNA was transiently increased in the spleen of WT, STAT1 KO and STAT1/RAG1 DKO mice on day 3 postinfection and was undetectable at day 7 postinfection. Moreover, IFN-β mRNA in infected STAT1 KO mice was significantly greater than that in WT mice but was 1.5-fold lower than that in STAT1/RAG1 DKO mice. In RAG1 KO mice, IFN-β mRNA was significantly increased on day 7 postinfection and was undetectable on day 35 postinfection. In the liver, IFN-β mRNA was low or undetectable in LCMV-infected WT and RAG1 KO mice at any observation time postinfection. By contrast, livers of infected STAT1 KO and STAT1/RAG1 DKO mice had significantly increased IFN-β mRNA levels on day 3 postinfection, which then subsided on day 7 postinfection and in STAT1/RAG1 DKO mice even further on day 35 postinfection.

In the plasma, IFN-α and -β were low or undetectable in mock-infected mice, irrespective of genotype (Fig 5B). Following infection, WT mice showed a small and transient increase of IFN-α levels on days 2 and 3 postinfection, while IFN-β was not detected. In RAG1 KO mice, neither IFN-α or IFN-β were detectable following infection any timepoint analyzed. By contrast, LCMV-infected STAT1 KO and STAT1/RAG1 DKO mice had a significant increase of IFN-α and IFN-β on day 3 postinfection, which returned to the mock-infected level at day 7 postinfection. Interestingly, on day 3 postinfection IFN-β levels were significantly higher in STAT1/RAG1 DKO mice than in STAT1 KO mice.

Taken together, these findings indicate that RAG1-deficiency in LCMV-infected STAT1 KO mice did not impair the local or systemic production of IFN-Is following LCMV infection. Further, IFN-β production in LCMV-infected STAT1/RAG1 DKO mice was exaggerated when compared with infected STAT1 KO mice, indicating that survival of the STAT1/RAG1 DKO mice was not due to impaired IFN-I production.

### Adaptive immune cells are required for the heightened systemic cytokine and chemokine response in LCMV-infected STAT1 KO mice

**Fig 6:**
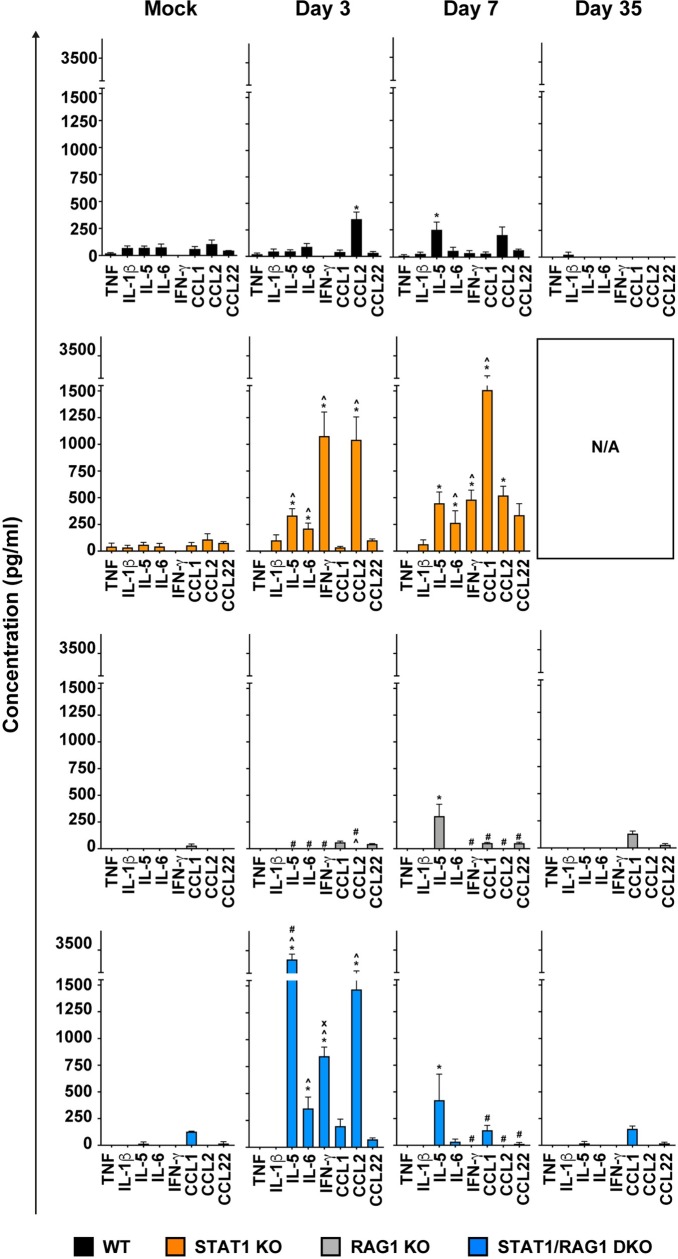
Adaptive immune cells are responsible for the prolonged and exaggerated systemic cytokine and chemokine production in the LCMV-infected STAT1 KO mice. Multiplex ELISA was performed on the plasma of LCMV-infected WT, STAT1 KO, RAG1 KO and STAT1/RAG1 DKO mice to determine the systemic levels of 8 distinct cytokines and chemokines and combined results from two independent experiments (n = 4–8 per time-point per genotype) are shown as the mean ± SEM. For significance (one-way ANOVA with Tukey post-test): *, P<0.05 compared with mock-infected mice; ^, P<0.05 compared with WT mice at the respective time points; #, P<0.05 compared with STAT1 KO mice at the respective time points; x, P<0.05 compared with RAG1 KO mice at the respective time points. No data was collected for LCMV-infected STAT1 KO mice on day 35 postinfection as none survived.

A hallmark of the disease in LCMV-infected STAT1 KO mice are heightened systemic cytokine and chemokine responses [23]. To delineate the roles of innate *versus* adaptive immune cells in this response, we quantified eight cytokines and chemokines that we identified previously to be differentially regulated in LCMV-infected STAT1 KO mice [23] in the plasma of mock- and LCMV-infected mice (Fig 6). Consistent with previous findings [22, 23], LCMV-infected WT mice showed a significant increase of CCL2 on day 3 postinfection and IL-5 on day 7 postinfection, only, while TNF, IL-1β, IL-6, IFN-γ, CCL1 and CCL22 levels did not change following infection (Fig 6). Similar to WT mice, LCMV-infected RAG1 KO mice showed a significant increase of IL-5 on day 7 postinfection. None of the other cytokines or chemokines changed at any day postinfection compared with mock-infected mice. By contrast, in LCMV-infected STAT1 KO mice, IL-5, IL-6, IFN-γ and CCL2 were increased significantly on day 3 postinfection and on day 7 postinfection, IL-5, IFN-γ and CCL2 remained elevated (Fig 6). Additionally, there was a significant increase of CCL1 in these mice on day 7 postinfection. Although similarly infected STAT1/RAG1 DKO mice showed a significant increase of IL-5, IL-6, IFN-γ and CCL2 on day 3 postinfection, with IL-5 being significantly greater (~10-fold) when compared with STAT1 KO mice, all cytokines except for IL-5 declined to the basal levels at day 7 postinfection. By day 35 postinfection, IL-5 had returned to the mock-infected level in infected STAT1/RAG1 DKO mice as well.

In all, these findings showed that in the absence of adaptive immune cells, the heightened systemic cytokine (IL-5, IL-6 and IFN-γ) and chemokine (CCL1 and CCL2) responses in LCMV-infected STAT1/RAG1 DKO mice only occurred in the early stage of disease. This demonstrates that adaptive immunity is essential for the prolonged inflammatory state in these mice. These findings also indicate there is a non-T-cell source for the production of IL-5 and point to an inhibitory role of adaptive immune cells in the production of this cytokine in the absence of STAT1 following LCMV infection.

### Adaptive immune cells are required for the prolonged, local overexpression of pro-inflammatory cytokine and chemokine genes in STAT1 KO mice during LCMV infection

**Fig 7:**
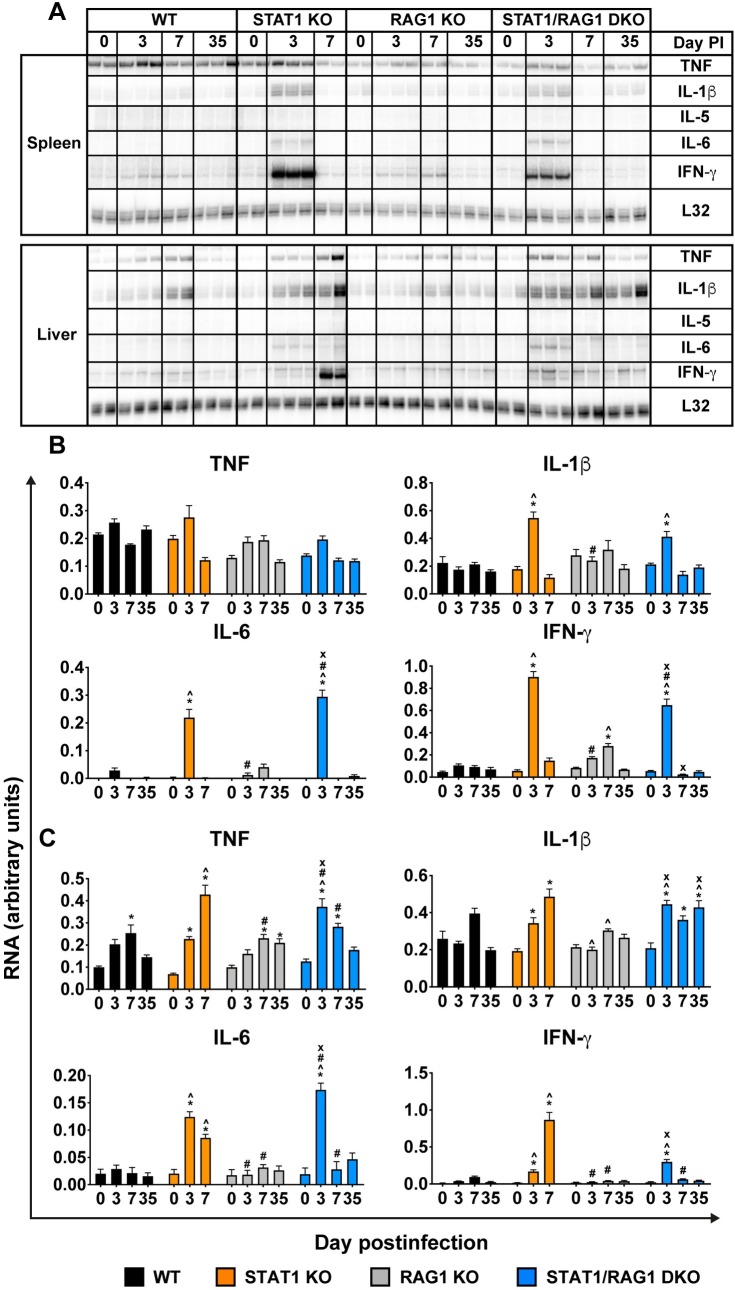
Adaptive immune cells are necessary for the prolonged and exaggerated expression of IL-6 and IFN-γ mRNAs in infected organs of LCMV-infected STAT1 KO mice. RPA was performed on total RNA (20 μg) of spleen (A and B) and liver (A and C) of WT, STAT1 KO, RAG1 KO and STAT1/RAG1 DKO mice infected i.p. with LCMV, to determine the levels of TNF, IL-1β, IL-6 and IFN-γ mRNA. Representative autoradiograph (n = 2–3 per time-point per genotype) is shown (A) and values from densitometric analysis were normalized to the corresponding L32 loading control and the combined results from two independent experiments (n = 4–6 per time-point per genotype) expressed as the mean ± SEM (B and C). For significance (One-Way ANOVA with Tukey post-test): *, P<0.05 compared with mock-infected mice; ^, P<0.05 compared with WT mice at the respective time-points; #, P<0.05 compared with STAT1 KO mice at the respective time-points; x, P<0.05 compared with RAG1 KO mice at the respective time-points. Day 0 PI denotes mock-infected control group.

In the previous experiment, we found that adaptive immune cells are required for the prolonged, heightened, systemic cytokine and chemokine responses in STAT1 KO mice following LCMV infection (Fig 6). To better understand the roles of innate *versus* adaptive immunity in the regulation of pro-inflammatory cytokine gene expression in infected tissues, we determined the levels of TNF, IL-1β, IL-5, IL-6 and IFN-γ mRNAs in spleen and liver (Fig 7). The TNF, IL-1β, IL-6 and IFN-γ mRNAs were low or undetectable in the spleen and liver of mock-infected mice, with no differences in the levels of these mRNAs between the four genotypes of mice.

In spleens from WT mice, there were no significant changes following LCMV infection in any of the cytokine mRNAs studied. Similarly, in spleens from LCMV-infected RAG1 KO mice, there were no significant changes for TNF, IL-1β and IL-6 mRNAs post infection. However, IFN-γ mRNA was significantly increased in these mice on day 7 postinfection, followed by a decline to the mock-infected level at day 35 postinfection. By contrast, LCMV-infected STAT1 KO and STAT1/RAG1 DKO mice had a significant increase of IL-1β, IL-6 and IFN-γ mRNAs but not TNF mRNA on day 3 postinfection, followed by a decline to the mock-infected levels at day 7 postinfection and the transcripts remained low on day 35 postinfection in STAT1/RAG1 DKO mice.

In the liver, there was a significant increase of TNF mRNA in WT and RAG1 KO mice on day 7 postinfection followed by a decline to the mock-infected level on day 35 postinfection in WT mice while the transcript remained elevated in RAG1 KO mice. In STAT1 KO and STAT1/RAG1 DKO mice, TNF mRNA was significantly increased above the mock-infected level at day 3 postinfection, followed by a further increase in STAT1 KO mice on day 7 postinfection and progressive decline in STAT1/RAG1 DKO mice at days 7 and 35 postinfection. No significant changes in IL-1β, IL-6 and IFN-γ mRNA levels were observed in the liver of WT and RAG1 KO mice following LCMV infection. By contrast, there was a significant increase of IL-1β mRNA in LCMV-infected STAT1 KO and STAT1/RAG1 DKO mice on day 3 postinfection and the transcript remained elevated at day 7 postinfection in STAT1 KO mice and at days 7 and 35 postinfection in STAT1/RAG1 DKO mice. IL-6 and IFN-γ mRNAs were significantly increased in LCMV-infected STAT1 KO and STAT1/RAG1 DKO mice on day 3 postinfection and while the transcripts remained elevated in STAT1 KO mice on day 7 postinfection (in the case of IFN-γ mRNA, a further 4-fold increase), they declined to the mock-infected levels in STAT1/RAG1 DKO mice at days 7 and 35 postinfection. IL-5 mRNA was undetectable in the spleen and liver of all genotypes of mice at any observation time pre or postinfection (Fig 7). Taken together, these results showed that the adaptive immune cells are required for the overexpression of *Il6* and *Ifng* genes in the liver of LCMV-infected STAT1 KO mice in the late stage of disease.

**Fig 8:**
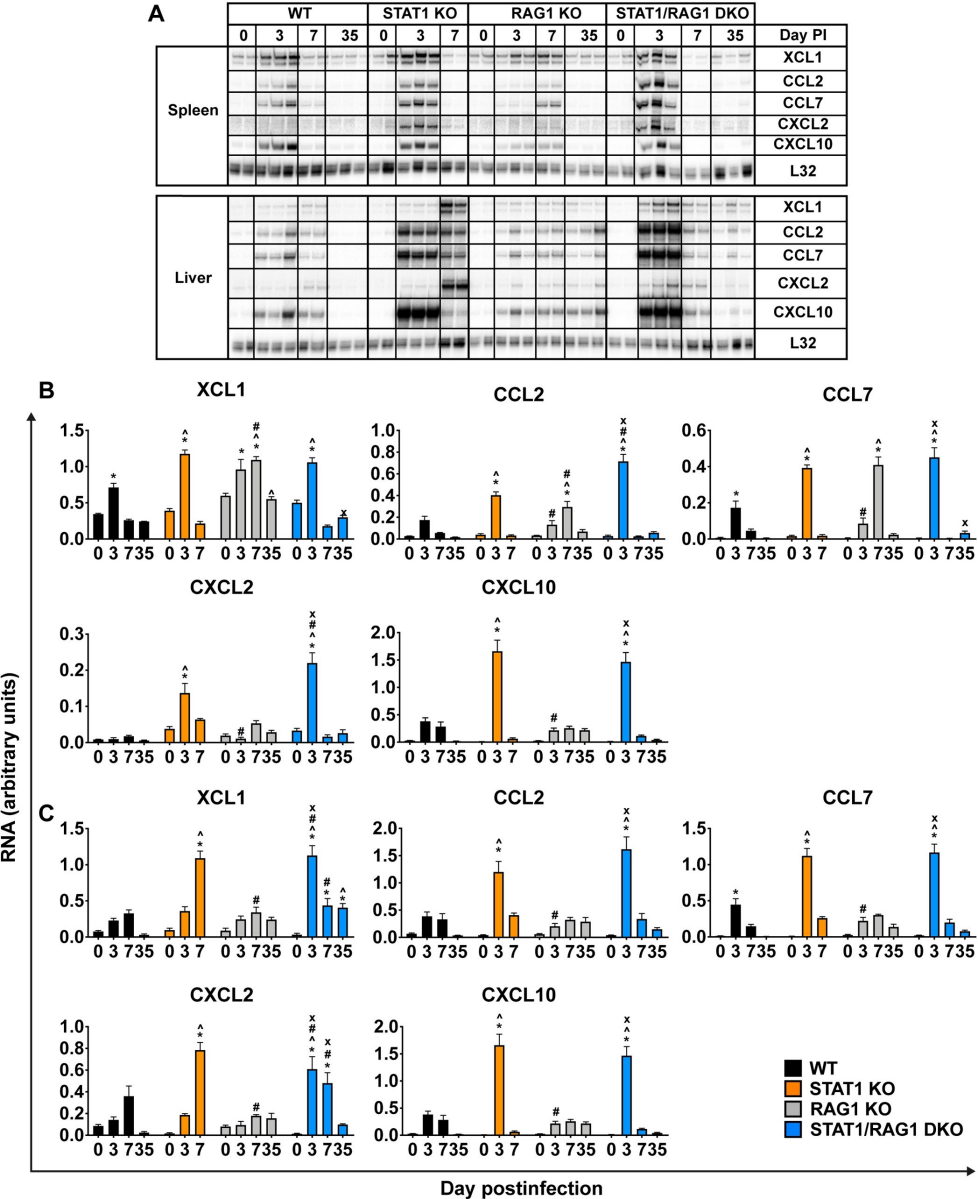
Adaptive immune cells are required for the prolonged and exaggerated expression of XCL1 and CXCL2 mRNAs in LCMV-infected STAT1 KO mice. RPA was performed on total RNA (20 μg) of spleen (A and B) and liver (A and C) of WT, STAT1 KO, RAG1 KO and STAT1/RAG1 DKO mice infected i.p. with LCMV, to determine the levels of XCL1, CCL2, CCL7, CXCL2 and CXCL10 mRNA. Representative autoradiograph (n = 2–3 per time-point per genotype) is shown (A) and values from densitometric analysis were normalized to the corresponding L32 loading control and the combined results from two independent experiments (n = 4 per time-point per genotype) expressed as the mean ± SEM (B and C). For significance (One-Way ANOVA with Tukey post-test): *, P<0.05 compared with mock-infected mice; ^, P<0.05 compared with WT mice at the respective time-points; #, P<0.05 compared with STAT1 KO mice at the respective time-points; x, P<0.05 compared with RAG1 KO mice at the respective time-points. Day 0 PI denotes mock-infected control group.

We next determined the levels of XCL1, CCL2, CCL7, CXCL2 and CXCL10 mRNAs in the spleen and liver of LCMV-infected WT, STAT1 KO, RAG1 KO and STAT1/RAG1 DKO mice. In the spleen and liver of mock-infected mice The XCL1, CCL2, CCL7, CXCL2 and CXCL10 mRNAs were low or undetectable, with no differences in the levels of these transcripts between the different genotypes (Fig 8). Following infection, only XCL1 and CCL7 mRNAs were significantly increased in the spleen of WT mice on day 3 postinfection, which declined to the mock-infected levels on day 7 postinfection and remained low at day 35 postinfection. In the spleen of RAG1 KO mice on day 3 postinfection, XCL1 mRNA was significantly increased and remained elevated at day 7 postinfection, followed by a decline to the mock-infected level on day 35 postinfection. Both CCL2 and CCL7 mRNAs were also significantly elevated in these mice on day 7 postinfection, followed by a decline to the mock-infected levels at day 35 postinfection. In STAT1 KO and STAT1/RAG1 DKO mice, XCL1, CCL2, CCL7, CXCL2 and CXCL10 mRNAs were significantly increased on day 3 postinfection, to levels that were significantly greater than in WT mice, followed by a decline to the mock-infected levels at day 7 postinfection, which remained low at day 35 postinfection in STAT1/RAG1 DKO mice. The maximal levels of CCL2 and CXCL2 mRNAs in STAT1/RAG1 DKO mice were significantly higher than in STAT1 KO mice.

In the liver of WT mice, there was a significant increase of CCL7 mRNA on day 3 postinfection, which declined to the mock-infected level at days 7 and 35 postinfection. Although there was a modest increase of XCL1, CCL2, CXCL2 and CXCL10 mRNAs in these mice following infection, this was not statistically significant. There were no significant changes of these transcripts in the liver of RAG1 KO mice at any time postinfection. By contrast, in STAT1 KO mice, XCL1 and CXCL2 mRNAs were significantly increased only on day 7 postinfection, while CCL2, CCL7 and CXCL10 mRNAs were significantly increased on day 3 postinfection, followed by a decline to the mock-infected level by day 7 postinfection. Similar to STAT1 KO mice, in STAT1/RAG1 DKO mice livers showed a significant increase of CCL2, CCL7 and CXCL10 mRNAs on day 3 postinfection, which subsequently declined to the mock-infected levels. However, unlike in STAT1 KO mice, livers of STAT1/RAG1 DKO mice showed a significant increase of XCL1 and CXCL2 mRNA on day 3 postinfection, which declined but remained elevated on day 7 postinfection. By day 35 postinfection, XCL1 mRNA remained elevated while CXCL2 mRNA declined to the mock-infected level. On day 7 postinfection, the levels of XCL1 and CXCL2 mRNAs in the livers of STAT1 KO mice were significantly higher than in STAT1/RAG1 DKO mice.

Collectively, these findings indicated that adaptive immune cells were dispensable for the overexpression of chemokine mRNAs (CCL2, CCL7, CXCL10) in the spleen and liver of LCMV-infected STAT1 KO mice. While the expression of XCL1 and CXCL2 mRNAs was significantly enhanced in the absence of adaptive immune cells in the liver of these mice on day 3 postinfection, on day 7 postinfection, adaptive immune cells were required for the heightened expression of these transcripts and paralleled the degree of leukocyte extravasation in these mice.

## Discussion

Studies using mice deficient for STAT1 have illuminated the central role that STAT1 plays in determining the outcome of viral infection. We have previously established that following infection with LCMV, STAT1-deficient mice develop a lethal, immunopathological disease [22, 23]. However, the relative contribution of the innate versus adaptive immune compartments in this disease remained unclear. Hence, in the current study, we used a genetic model of RAG1-deficiency, in which adaptive immune cells are non-functional [24]. Our findings show that STAT1 has extensive involvement in both innate and adaptive immunity–lessening both the early, inflammatory responses of innate immunity and the sustained, destructive actions of adaptive immunity.

Our results demonstrate that LCMV infection causes a biphasic disease in STAT1 KO mice. The first phase is characterized by exaggerated production of cytokines and chemokines, including IFN-Is and is associated with thrombocytopenia and rapid weight loss. These changes are non-lethal and independent of adaptive immunity since in the absence of T and B cells, STAT1/RAG1 DKO mice survived LCMV infection. The initial phase is then followed by a second phase, which is dependent on adaptive immunity and characterized by sustained cytokine and chemokine production, systemic neutrophilia, continually excessive leukocyte extravasation into peripheral organs, progressive weight loss and ultimately, death of these mice.

Increased virus RNA levels in the absence of STAT1 but not RAG1 on day 3 postinfection suggests that during the early stages of infection, IFN-Is rather than adaptive immune cells are critical for inhibiting initial virus replication and spread. Accordingly, increased virus replication and spread is seen in the absence of the IFN-I receptor [19]. Of note and in contrast to a previous report [29], RAG1-deficiency did not impair IFN-I responses or dynamics following LCMV infection. A possible explanation for this dichotomy between our findings and those of Louten *et al*. could be differences in the virus dose used, which was 40-fold lower in our study [29]. Notably, we found that viral load was low or undetectable in RAG1 KO mice on days 3 and 7 postinfection, similar to the findings in LCMV-WE-infected RAG2 KO mice [30]. Müller *et al*. argued that because LCMV has early tropism for splenic marginal zone, disrupted splenic microarchitecture in RAG2 KO mice results in lack of early proliferation of the virus [30]. However, in our study, increased viral RNA levels in STAT1/RAG DKO mice compared with the RAG1 KO mice in the early days of infection suggests that in RAG1 KO mice, there is STAT1-dependent, likely IFN-I-dependent, robust, early restriction of viral replication. In contrast to the early timepoints postinfection, during late stages of infection, LCMV RNA levels were increased in the absence of RAG1, independent of STAT1. This is in line with similar findings that had demonstrated a critical requirement of adaptive immune cells, especially CD8^+^ T cells, to restrict and clear LCMV in WT mice [20, 21]. Interestingly, the degree of LCMV replication and spread was similar in liver and kidney of STAT1/RAG DKO mice when compared with STAT1 KO mice, suggesting that there is little contribution of adaptive immune cells in restricting viral replication in STAT1-deficient mice. The IFN-Is directly promote the clonal expansion, survival, production of IFN-γ and development of cytotoxic functions of CD8^+^ T cells [7]. During influenza virus infection, STAT1-deficient mice have defective CD8^+^ T cell activation, expansion and survival [31]. Similarly, IFN-I responses are required for accumulation and effector function of LCMV-specific CD8^+^ T cells [19]. Although not determined in our study, given the published evidence of a critical requirement for IFN-I and STAT1 for functionality of CD8^+^ T cells, it is likely that LCMV-infected STAT1 KO mice have dysfunctional CD8^+^ T cells. However, our previous finding that depletion of CD8^+^ cells does not rescue LCMV-infected STAT1 KO mice [23] argues against a critical role for CD8^+^ T cells in the second phase of disease. Interestingly, lethal disease was also observed in STAT1 KO mice infected with a low dose of the immunosuppressive strain, LCMV clone 13 (S3 Fig). In contrast to liver and kidney, there was significantly more LCMV-NP RNA in STAT1/RAG1 DKO mice than in STAT1 KO mice in the spleen and lung. While this indicates possible organ-specific, inhibitory actions of adaptive immune cells on viral replication and spread in STAT1-deficient mice, it remains to be clarified.

Our findings confirmed previous reports of exaggerated production of IFN-Is in LCMV-infected STAT1 KO mice compared with WT mice [22, 23]. Moreover, we had demonstrated that IFN-I production and signaling is necessary for the lethal disease in LCMV-infected STAT1 KO mice [22]. Our findings here, showed that LCMV-infected STAT1 KO and STAT1/RAG1 DKO mice had similar systemic levels of IFN-α and -β and local tissue IFN-β mRNA, suggesting that IFN-I production in LCMV-infected STAT1 KO mice is regulated largely by innate immunity, while adaptive immunity is dispensable. In addition to increased IFN-I levels, LCMV infection in the absence of STAT1 resulted in increased levels of pro-inflammatory cytokines, including IL-6, IFN-γ and IL-5, which have previously been suggested to contribute to death in LCMV-infected STAT1 KO mice [23]. In contrast to IFN-γ, which is not required for the lethal disease in LCMV-infected STAT1 KO mice [23], it is conceivable that IL-6 contributes to this disease. For example, in a systemic inflammatory model of sepsis, IL-6 has been shown to be deleterious [32]. The high IL-5 levels seen in STAT1 KO and STAT1/RAG DKO mice may contribute to early weight loss and more severe thickening of the basement membrane of bronchial epithelial cells, which was exaggerated in STAT1/RAG1 DKO mice compared with STAT1 KO mice following infection. Indeed, anti-IL5 antibody treatment inhibits the induction of airway subepithelial fibrosis in a murine model of atopic asthma, suggesting a pathogenic role for this cytokine in lung pathology [33]. However, it seems unlikely that IL-5 has an instrumental role in the lethality of LCMV-infected STAT1 KO mice since STAT1/RAG1 DKO mice had greater IL-5 levels than STAT1 KO mice following infection, suggesting that adaptive immune cells inhibit the production of IL-5. Notwithstanding, the precise role of IL-5 in the pathological context of LCMV-infected STAT1 KO mice needs to be clarified further in future studies.

Arenavirus infection can cause severe hemorrhagic diseases in humans [25]. Similarly, Misumi and colleagues described a mouse model where infection with LCMV causes gross thrombocytopenia, vascular leakage and death in the B6/PL strain of mice [28]. Baccala and colleagues made similar observations in LCMV-Cl13 infected New Zealand Black mice [27] showing that hemorrhage required IFN-I signaling in nonhematopoietic cells. In our study, we found that in the absence of STAT1, LCMV infection resulted in severe thrombocytopenia, which was independent of adaptive immune cells. This is consistent with previous reports showing that IFN-I can induce thrombocytopenia directly [34, 35]. However, unlike Misumi *et al*. and Baccala *et al*. [27, 28], we did not find evidence of gross vascular leakage or severe hemorrhaging in LCMV-infected STAT1 KO, evidenced by the lack of significant anemia and overt bleeding. It remains to be clarified if these differences are due to the virus strain used–Misumi *et al*. and Baccala *et al*. used high dose infection with the immunosuppressive strain LCMV clone 13 –or if STAT1-dependent IFN-I signaling is required for extensive vascular disruption to occur.

The greater systemic neutrophilia and neutrophil migration into the liver of LCMV-infected STAT1 KO mice when compared with STAT1/RAG1 DKO mice was consistent with the observed differences in the levels of neutrophil chemoattractants CXCL2, CCL1 and CCL2. Although these findings showed a correlation between the degree of neutrophilia and severity of disease in these mice, we could not clearly delineate the potential pathological roles of neutrophils in this model. Our attempts to ablate neutrophils using anti-Ly6G or anti-Gr-1 antibody injection, commonly used approaches, had limited success (S4 Fig). Yet, it is noteworthy that 20% reduction in neutrophils in infected STAT1 KO mice had no effect on body weight and survival (S4 Fig), arguing against neutrophils being the primary mediators of lethality. Instead, in our model, neutrophils may be indicators of the severity of disease, as in several cases of systemic inflammation (reviewed in [36]).

In the absence of STAT1, LCMV infection results in a distinctive, biphasic disease. The first innate immunity-driven phase of disease is characterized by rapid weight loss, thrombocytopenia, systemic cytokine and chemokine responses and leukocyte infiltration of various tissues. The second adaptive immunity-driven phase of disease shows continued cytokine and chemokine production, persistent leukocyte extravasation into infected tissues and leads, ultimately, death of the host. However, when the adaptive immune response is absent, the first phase of disease largely resolves resulting in survival of the infected host. Taken together, these findings demonstrate extensive involvement of STAT1 in both innate and adaptive immunity.

## Materials and methods

### Mice

STAT1 KO mice [37] were originally provided by Dr. Joan Durbin and a breeding colony maintained at the University of Sydney. RAG1 KO mice [24] were obtained from Animal Resources Centre (ARC; Canning Vale, Australia). STAT1/RAG1 DKO mice were produced by interbreeding, and the genotype verified by PCR analysis of tail DNA and by flow cytometric analysis of tail vein blood, by confirming the lack of CD4^+^ and CD8^+^ T cells. All mice used were on the C57BL/6 background and were housed in specific pathogen-free conditions in the animal facility of the University of Sydney.

### Ethics statement

Ethics approval for all animal experiments was obtained from the animal ethics committee of The University of Sydney (AEC 1056/16). All animal experiments were performed in compliance with the NSW Animal Research Act and its associated regulations and the 2004 NHMRC ‘Australian code of practice for the care and use of animals for scientific purposes’. Euthanasia of mice was performed by CO_2_ or isoflurane inhalation followed by decapitation.

### LCMV infection

All mice were 8–16 weeks old at the time of infection and were age- and sex-matched in all experiments. The LCMV Armstrong 53b stock was obtained originally from a triple plaque-purified clone that was subsequently passaged twice in BHK cells [38]. For virus inoculation, mice were given i.p. injection of 500 PFU LCMV-ARM 53b in 200 μl of phosphate buffered saline (PBS) plus 2.5% fetal bovine serum (FBS). Mock-infected mice received the same volume of PBS plus 2.5% FBS without the virus.

### RNase Protection Assay (RPA)

Total RNA was prepared from snap frozen tissue using TRI Reagent (Sigma-Aldrich, Castle Hill, Australia) according to the manufacturer’s instructions. RPA was performed using specific riboprobes as described previously [39–41]. The bands were densitometrically quantified using ImageJ software [42]. The intensity of each target RNA band was normalized to that of the loading control, L32 [43].

### Hematology

Mice were deeply anaesthetized by halothane inhalation and euthanized by exsanguination via cardiac puncture. A one-tenth volume of 0.5 M EDTA was added to collected blood to prevent coagulation and blood smears were prepared. Routine Diff-Quik stain of blood smears were performed at the Histopathology Core Facility (Department of Pathology, the University of Sydney). Stained smears were examined under a DM4000B microscope (Leica, Wetzlar, Germany). Bright field images were acquired using a SPOT Flex 15.2 64 Mp Shifting Pixel camera, and SPOT Advanced 4.5 software (Diagnostic Instruments). To determine hematological parameters, blood was analyzed using an XP-100 hematology analyzer (Sysmex, Kobe, Japan).

### Histology

Once removed, organs were placed immediately in PBS-buffered 4% paraformaldehyde (PFA, pH 7.4; Sigma-Aldrich) for 24h at 4°C and were subsequently embedded in paraffin. Histological analysis was performed on 5–8 μm thick sections of paraffin-embedded tissue. Routine histology (hematoxylin and eosin (H&E)) of tissue sections was performed at the Histopathology Core Facility (Department of Pathology, University of Sydney). Stained sections were examined under a DM4000B bright field microscope (Leica, Wetzlar, Germany). Bright field images were acquired using a SPOT Flex 15.2 64 Mp Shifting Pixel camera, and SPOT Advanced 4.5 software (Diagnostic Instruments).

### Leukocyte isolation from spleen and liver

On day 7 postinfection, mice were deeply anaesthetized by halothane inhalation and euthanized by exsanguination via cardiac puncture and the mice were perfused with sterile PBS. The spleens were homogenized and passed through a 70 μm cell strainer. After washing the strainer with PBS, cells were centrifuged at 460 x *g* for 10 minutes at 4°C and washed in fluorescence-activated cell sorting (FACS) buffer (5% FBS, 5 mM EDTA, PBS (pH 7.4)). Livers were chopped with razor blades and incubated with 20 ml per whole liver in digestion buffer (100 U/ml Collagenase type IV and 10 U/ml DNase I type IV in Dulbecco’s modified Eagle’s medium) at 37°C for 1 h with gentle agitation every 15 minutes. Tissue fragments were dispersed and passed through a 70 μm cell strainer. After washing the strainer with FACS buffer, cells were centrifuged at 460 x *g* for 15 minutes at 4°C. The cells were then suspended in 30% Percoll (GE Healthcare, Castle Hill, NSW, Australia) and layered over 80% Percoll. Samples were centrifuged at 1,830 x *g* for 25 minutes with no brake at room temperature, and cells at the interface were collected and washed in FACS buffer.

### Flow cytometry

Single-cell-suspensions of splenocytes and liver leukocytes were stained for cell surface markers using specific fluorophore-conjugated antibodies optimized for flow cytometry. Reagents and antibodies used in this study were LIVE/DEAD Fixable Blue Dead Cell Stain (ThermoFisher), CD11b-BUV395 (Clone: M1/70; BD Biosciences), Siglec-F-BUV615 (Clone: E50-2440; BD Biosciences), NK1.1-BUV661 (Clone: PK136; BD Biosciences), B220-BUV737 (Clone: RA3-6B2; BD Biosciences), CD8α-BUV805 (Clone: 53–6.7; BD Biosciences), MHCII(I-A/I-E)-BV510 (Clone: M5/114.15.2; BioLegend), CD4-BV570 (Clone: RM4-5; BioLegend), SCA-1-BV711 (Clone: D7; BioLegend), CD11c-BV785 (Clone: N418; BioLegend), Ly6C-FITC (Clone: HK1.4; BioLegend), Siglec-H-PerCP/Cy5.5 (Clone: 551; BioLegend), F4/80-PE (Clone: BM8; BioLegend), CD3ε-PE/Cy5 (Clone: 145-2C11; ThermoFisher), CD80-PE/Cy7 (Clone: 16-10A1; BioLegend), CD115-AF594 (Clone: AFS98; BioLegend), CCR2-APC (Clone: 475301; R&D Systems), CD45-AF700 (Clone: 30-F11; BioLegend), CD48-APC/Cy7 (Clone: HM48-1; BioLegend), Ly6G-Biotin (Clone: 1A8; BioLegend; with secondary antibody streptavidin-DyLight 800; ThermoFisher). Stained and fixed (4% PFA; 10 min in the dark) cells were analyzed with a Becton Dickson custom 10-laser ‘LSR-II’ flow cytometer and FlowJo software (v.10.4.1).

### Cytokine and chemokine quantification

Enzyme-linked immunosorbent assay (ELISA) kits (InvivoGen) were used to measure IFN-α and IFN-β in the plasma following the manufacturer’s instructions. The plates were scanned with FLUOstar Omega microplate reader. Limits of detection: IFN-α, 7.8–500 pg/ml and IFN-β, 15.6–1000 pg/ml. A Q-plex array (Quansys Biosciences) was used to determine cytokine and chemokine levels in plasma. The plate was scanned with Odyssey Infrared Imaging System (LI-COR Biosciences) and analyzed with Q-View (Quansys Biosciences). Limits of detection: TNF, 4.12–3000 pg/ml; IL-1β, 19.2–14000 pg/ml; IL-5, 6.86–5000 pg/ml; IL-6, 6.86–5000 pg/ml; IFN-γ, 10.97–8000 pg/ml; CCL1, 5.6–4000 pg/ml; CCL2, 4.12–3000 pg/ml; CCL22, 5.6–4000 pg/ml.

### Statistical analysis

For the survival curve, statistical significance was calculated using Log-rank; Mantel-Cox test. P-value of less than 0.05 was considered significant. Statistical significance was calculated using two-way ANOVA (when there were equal number of timepoints postinfection per genotype) or one-way ANOVA (when there were disproportionate number of timepoints postinfection per genotype) of group data with Tukey’s post-test when assessing the differences in the levels of RNA and proteins and cell numbers between different genotypes of mice at different time postinfection. All statistical analyses were performed in Prism 7 software (GraphPad).

## Supporting information

S1 Fig Representative gating strategy for flow cytometric analysis of leukocytes from mock-infected WT spleen is shown. P1: CD8^+^ T cells, P2: CD4^+^ T cells, P3: B cells, P4: plasmacytoid dendritic cells (pDCs), P5: natural killer (NK) cells, P6: eosinophils, P7: Ly6G^int^ neutrophils, P8: Ly6G^hi^ neutrophils, P9: CD8α^+^ dendritic cells (DCs), P10: CD11b^+^ DCs, P11: F4/80^hi^ CD11b^lo^ macrophages, P12: immature granulocytes, P13: Ly6C^hi^ monocytes, P14: Ly6C^lo^ monocytes.
(TIF)

S2 Fig RAG1-deficiency causes disruption of splenic architecture independent of infection with LCMV. Representative H&E images of spleen. Scale bar = 250 μm. Representative images from 3 independent experiments are shown. No images were collected for LCMV-infected STAT1 KO mice on day 35 postinfection as none survived.
(TIF)

S3 Fig LCMV-Cl13-infected STAT1 KO mice succumb to lethal wasting disease. WT (n = 6) and STAT1 KO mice (n = 6) were infected with 1000 pfu of LCMV-Cl13 i.p. as described in Materials and Methods. (A) Weight changes postinfection. (B) Survival outcome. For significance (one-way ANOVA with Tukey post-test): *, P<0.05 for STAT1 KO mice compared with WT mice.
(TIF)

S4 Fig Anti-mouse Ly6G or Gr-1 antibody-mediated reduction of neutrophils does not rescue LCMV-infected STAT1 KO mice from lethal wasting disease. LCMV-infected STAT1 KO mice were injected with PBS (n = 8) or Ly6G antibody (500 μg) (n = 6) on one day prior to infection and days 2 and 5 postinfection. (A) Weight changes post-infection. Black arrow–antibody injection; Red arrow–virus inoculation (B) Percentage of neutrophils (SSC-A^hi^ CD11b^hi^ Ly6G^+^) in peripheral blood on day 7 postinfection, as determined by flow cytometric analysis. LCMV-infected STAT1 KO mice were injected with PBS (n = 5) or Gr-1 antibody (250 μg) (n = 6) on one day prior to infection and days 1, 3, 5 and 6 postinfection. (C) Weight changes post-infection. Black arrow: antibody injection; Red arrow: virus inoculation (D) Percentage of neutrophils in peripheral blood on day 7 postinfection, as determined by Sysmex XP-100. Bar and error bars represent mean ± SEM. For significance (Mann-Whitney U test): *, P<0.05 compared with PBS-injected mice.
(TIF)
