# Supplementary figures and images for: Contribution of STAT1 to innate and adaptive immunity during type I interferon-mediated lethal virus infection
Source: PLoS Pathog. 2020 Apr 20;16(4):e1008525. doi: 10.1371/journal.ppat.1008525 (PMC7192509; doi:10.1371/journal.ppat.1008525)

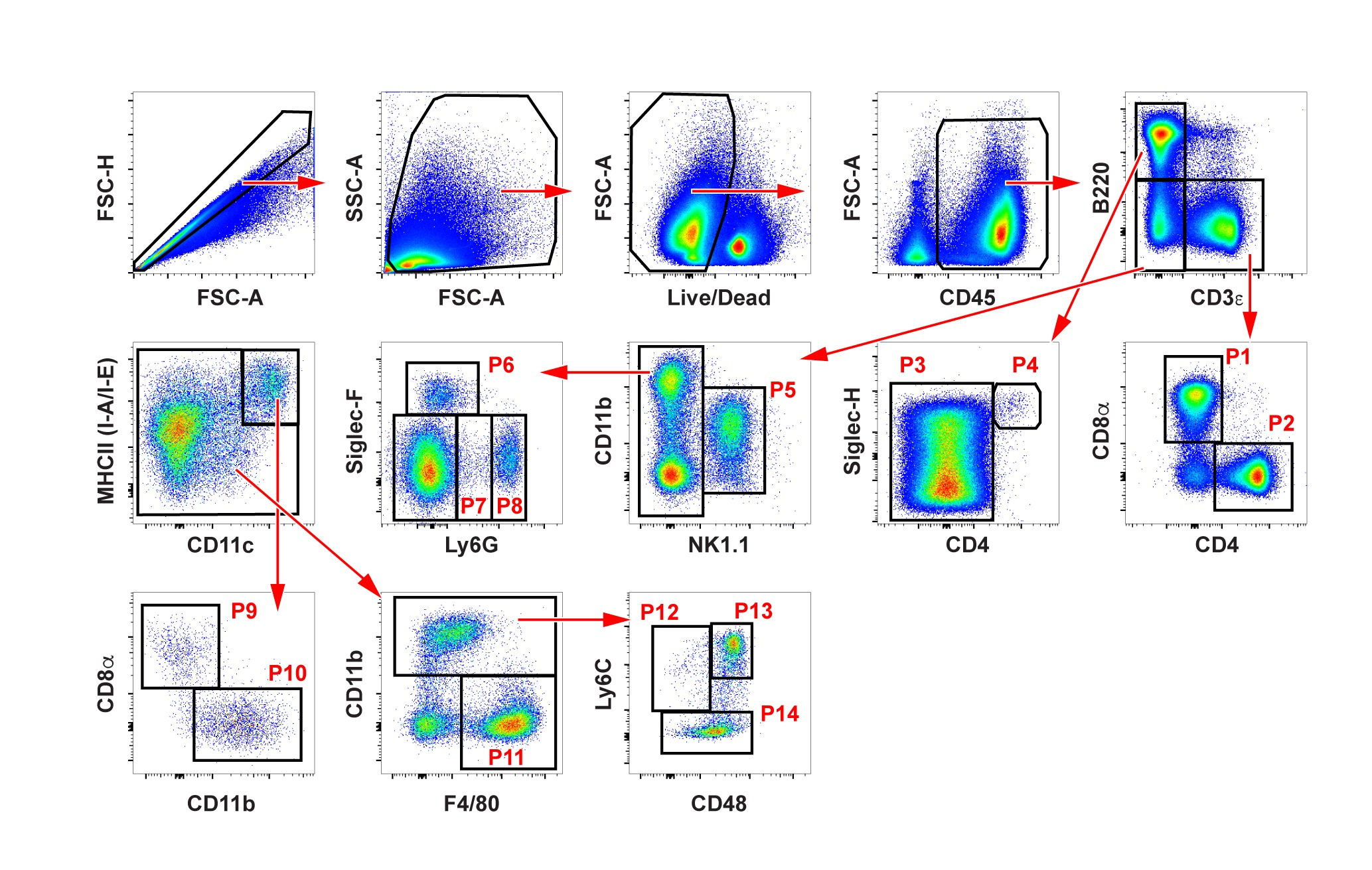

Supplement: S1 Fig — P1: CD8+ T cells, P2: CD4+ T cells, P3: B cells, P4: plasmacytoid dendritic cells (pDCs), P5: natural killer (NK) cells, P6: eosinophils, P7: Ly6Gint neutrophils, P8: Ly6Ghi neutrophils, P9: CD8α+ dendritic cells (DCs), P10: CD11b+ DCs, P11: F4/80hi CD11blo macrophages, P12: immature granulocytes, P13: Ly6Chi monocytes, P14: Ly6Clo monocytes. (TIF) [file ppat.1008525.s001.tif]

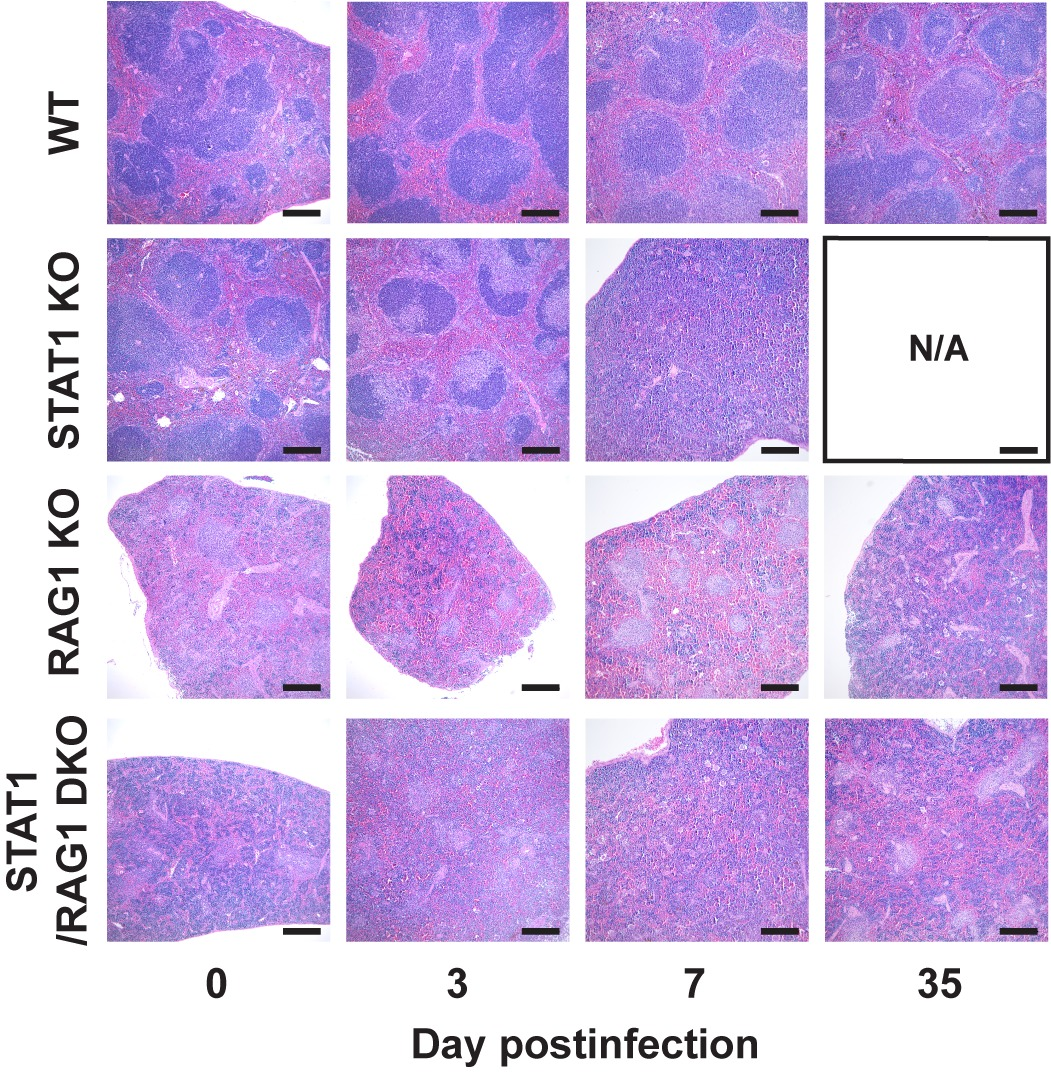

Supplement: S2 Fig — Representative H&E images of spleen. Scale bar = 250 μm. Representative images from 3 independent experiments are shown. No images were collected for LCMV-infected STAT1 KO mice on day 35 postinfection as none survived. (TIF) [file ppat.1008525.s002.tif]

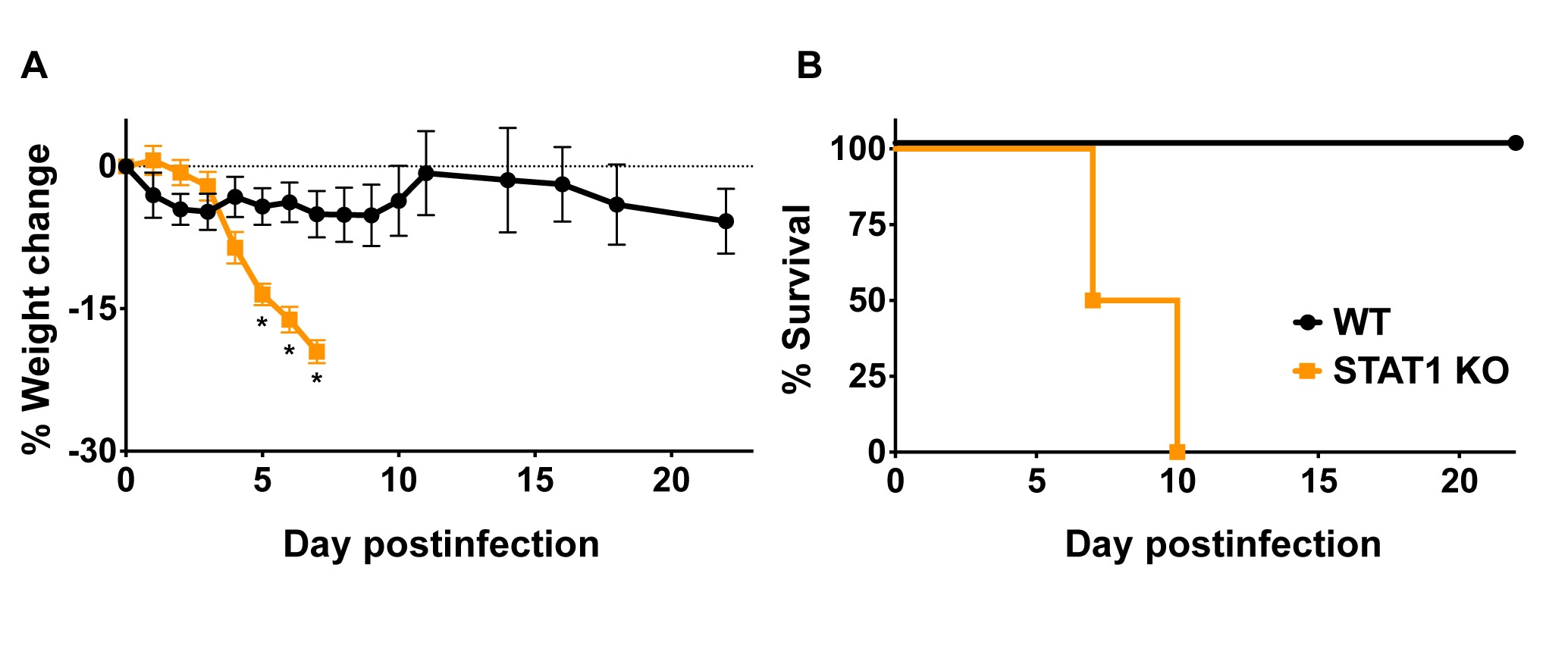

Supplement: S3 Fig — WT (n = 6) and STAT1 KO mice (n = 6) were infected with 1000 pfu of LCMV-Cl13 i.p. as described in Materials and Methods. (A) Weight changes postinfection. (B) Survival outcome. For significance (one-way ANOVA with Tukey post-test): *, P<0.05 for STAT1 KO mice compared with WT mice. (TIF) [file ppat.1008525.s003.tif]

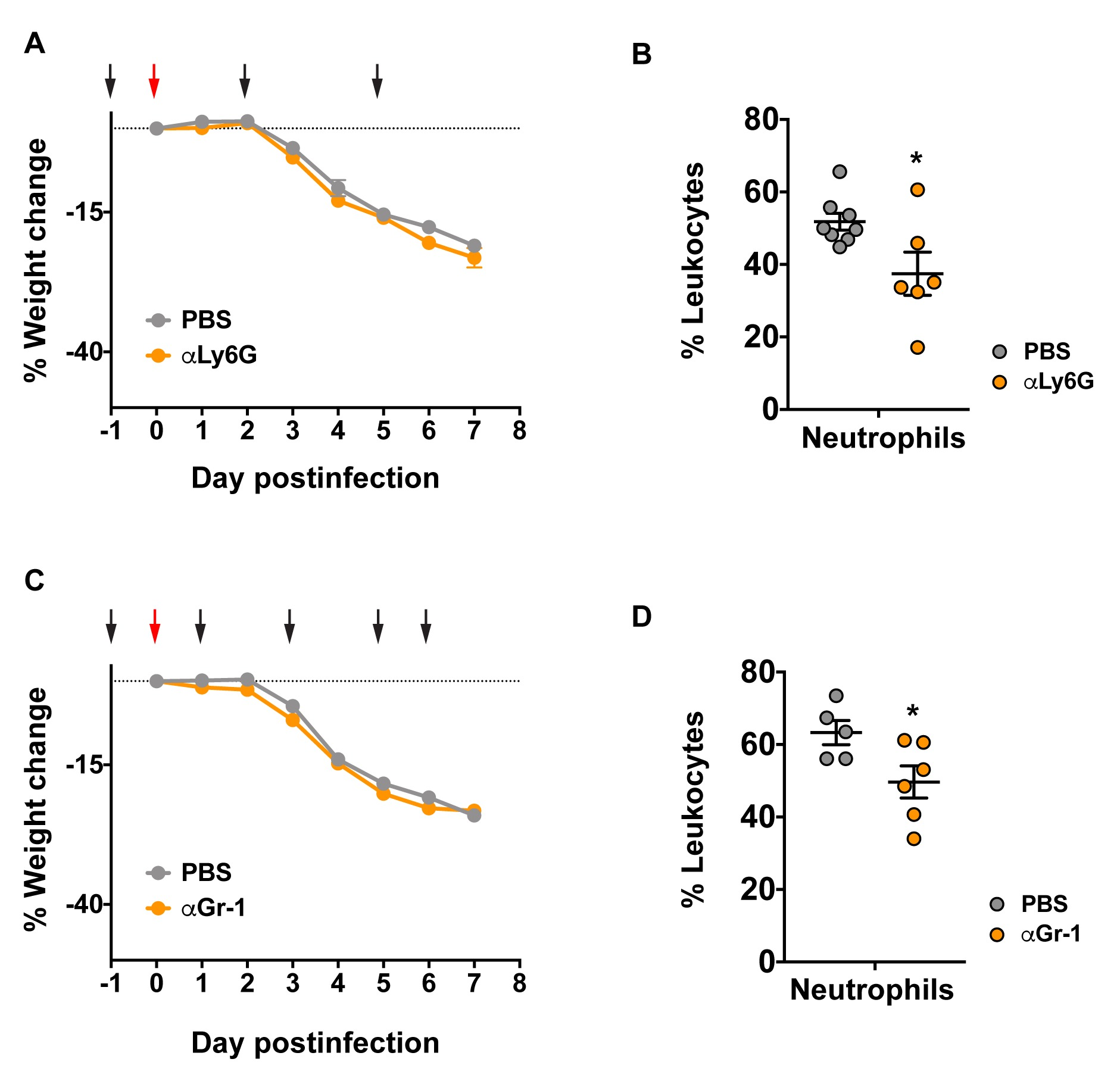

Supplement: S4 Fig — LCMV-infected STAT1 KO mice were injected with PBS (n = 8) or Ly6G antibody (500 μg) (n = 6) on one day prior to infection and days 2 and 5 postinfection. (A) Weight changes post-infection. Black arrow–antibody injection; Red arrow–virus inoculation (B) Percentage of neutrophils (SSC-Ahi CD11bhi Ly6G+) in peripheral blood on day 7 postinfection, as determined by flow cytometric analysis. LCMV-infected STAT1 KO mice were injected with PBS (n = 5) or Gr-1 antibody (250 μg) (n = 6) on one day prior to infection and days 1, 3, 5 and 6 postinfection. (C) Weight changes post-infection. Black arrow: antibody injection; Red arrow: virus inoculation (D) Percentage of neutrophils in peripheral blood on day 7 postinfection, as determined by Sysmex XP-100. Bar and error bars represent mean ± SEM. For significance (Mann-Whitney U test): *, P<0.05 compared with PBS-injected mice. (TIF) [file ppat.1008525.s004.tif]
